# Cytokine Effect of TLR3, TLR4, and TLR7 Agonists Alone or Associated with* Leishmania infantum* Antigen on Blood from Dogs

**DOI:** 10.1155/2018/5693736

**Published:** 2018-11-12

**Authors:** Pamela Martínez-Orellana, Sara Montserrat-Sangrà, Paulina Quirola-Amores, Noemí González, Laia Solano-Gallego

**Affiliations:** Departament de Medicina i Cirurgia Animals, Facultat de Veterinària, Universitat Autònoma de Barcelona, Bellaterra, Spain

## Abstract

Activation of toll-like receptors (TLRs) has been shown to play an important role in leishmaniosis by enhancing the parasite specific immune responses to control infection. However, the role of TLR agonists has not been studied in detail in dogs. The aim of this study was to determine the effect of TLR3, TLR4, and TLR7 agonists (TLR3a, TLR4a, and TLR7a) alone or in combination with* Leishmania infantum* antigen (LSA) on TNF-*α* and IL-6 production in blood from dogs living in endemic areas of canine leishmaniosis (CanL). Twenty-four healthy dogs from Catalonia (n=14) and Ibizan hound dogs from the island of Mallorca (n=10) were enrolled. Whole blood with TLR3a, TLR4a, and TLR7a alone or combined with LSA were cultured separately, and IFN-*γ*, TNF-*α*, and IL-6 were measured by ELISA. A significant increase of TNF-*α* was found for all conditions studied compared to medium alone. Stimulation with TLR4a* (p=0.0001) *and TLR7a* (p=0.005)* presented a significantly marked increase in TNF-*α* and IL-6 production compared to TLR3a. Importantly, significantly higher TNF-*α* production was found in LSA+TLR4a* (p=0.0001) *stimulated blood and LSA+TLR7a* (p=0.005)* compared to LSA alone. All dogs showed higher TNF-*α* production after LSA+TLR7a compared to TLR7a* (p=0.047) *and LSA+TLR3a compared to TLR3a* (p=0.052)*. These data indicate a marked inflammatory cytokine effect of TLR4a and TLR7a on blood from healthy dogs living in endemic areas of CanL. Additionally, LSA+TLR7a promoted a synergistic proinflammatory effect with TNF-*α* in all dogs. Those findings suggest an active role of TLRs in proinflammatory responses, which might be strongly involved in the process of disease resolution.

## 1. Introduction

Leishmaniosis are a group of protozoan diseases caused by several species of* Leishmania *(class Kinetoplastea, family Trypanosomatidae). More than 70 countries are endemic for* Leishmania *infection, including countries in the Mediterranean basin, the Middle East, Asia, Africa, and South America [1].* Leishmania infantum* is the species most commonly associated with canine infections [2]. In endemic Mediterranean areas, dogs are infected through the bite of sand flies of the genus* Phlebotomus *in the warmer months, from April to November [3–5].

Canine leishmaniosis (CanL) can be manifested with a range of clinical signs and laboratory abnormalities that include nonpruritic skin lesions such as exfoliative dermatitis and ulcerations, local or generalized lymphadenomegaly, weight loss, poor appetite, ocular lesions, epistaxis, lameness, renal failure, and diarrhea [6]. In addition, different degrees of disease severity exist [6]. Moreover, persistent subclinical infections in dogs are common in endemic areas such as the Mediterranean basin [7, 8].

Dogs of any breed are susceptible to* L. infantum *infection, but breeds such as Bulldogs, Dobermanns [9], Boxers, German Shepherds, and Rottweilers seem to be more susceptible to develop CanL [2]. However, Ibizan hounds are especially CanL-resistant to develop the disease living in endemic areas such as the island of Mallorca [10]. Mixed T helper 1 and T helper 2 (Th1/Th2) responses with a dominant Th1 profile are required by humans and dogs for protection against viscerotropic infection by* L. donovani* and* L. infantum* [11, 12]. Classically, Th1 responses are associated with resistance and Th2 responses are associated with susceptibility in dogs [13, 14], humans [15], and rodent models [16]. Moreover, it is demonstrated in both canine and murine models that Th1 cytokines as IFN-*γ* and TNF-*α* activate macrophages to kill* L. infantum* via increasing the nitric oxide (NO) pathway [17–19].

The cell-mediated immune (CMI) response is the major defence against CanL [20, 21] and is indispensable for the resolution of this parasitic infection [22, 23]. One clear example is observed in Th1 responses developed by CanL-resistance Ibizan hound [10]. In contrast, disease development and severity in CanL is often correlated with a marked humoral response and the abrogation of Th1 cytokines production [24, 25], which cannot control the infection.

Toll-like receptors (TLRs) are type 1 membrane proteins that belong to the group of patter recognition receptors (PRRs) [26]. Ten TLRs are found in dogs [27] and they bind conserved molecular structures found in large groups of pathogen-associated molecular patterns (PAMPs). TLRs are located in either plasma membrane or internal membranes mainly of macrophages, dendritic cells (DC), natural killer (NK) cells, and lymphocytes (T and B) and induce proinflammatory cytokines, type-1 IFN, chemokines, and costimulatory molecules [26, 28] as well as shaping adaptive immunity [29]. TLRs have been shown to play an important role in leishmaniosis [30] because they are one of the most important nonclonal sets of PRRs families [28].

There are several studies that have demonstrated the importance of TLR3 [31, 32], TLR4 [33], and TLR7 [34] in recognition, control, and protection against* Leishmania* infection. Those previous works are based mainly on investigations of* Leishmania major* in a murine model [30]. However, limited information is available about the function of TLRs in canine* L. infantum* infection [20].

Toll-like receptor agonists (TLRa) are natural and synthetic molecular structures (PAMPs)[35] that bind to TLRs with powerful immunostimulant potential. The ability of TLRa is to activate signalling pathways to manage innate and acquired immunity [36]. Therefore, they amplify parasite immune responses by stimulating the production of proinflammatory cytokines, which might play an important role in controlling* Leishmania* infection [37]. The current treatment of CanL is not sufficiently effective; thus the use TLR ligands as adjuvants for vaccine or as immunotherapy could be an interesting tool against CanL. Previously, TLR2 agonist [Pam3CSK4, a synthetic derivative of triacylated lipoproteins (TLR2a)] used alone was shown to enhance the production of the proinflammatory cytokines like TNF-*α* and IL-6 in dogs with clinical leishmaniosis, infected “resistant” and healthy noninfected. Furthermore, a combination of* L. infantum *soluble antigen (LSA)+TLR2a promoted a synergistic proinflammatory effect with TNF-*α* in Ibizan hounds [38]. The hypothesis of this study was that TLR3, TLR4, and TLR7 agonists (TLR3a, TLR4a, and TLR7a) alone will enhance the production of proinflammatory cytokines in canine* ex vivo* whole blood. In addition, we hypothesized that the combination of TLRa with LSA promotes a synergistic release of proinflammatory cytokines when compared with LSA or TLR ligands alone. Therefore, the main objective of the present study was to investigate and expand the knowledge on the effect of others TLRa different from TLR2a as TLR3a [Poly (I:C)], TLR4a [Monophosphoryl lipid A (MPLA)], and TLR7a [Imiquimod-R837 (IMQ)] alone or in combination with* L. infantum* antigen on* ex vivo* whole blood from apparently healthy dogs living in an endemic area of leishmaniosis (Catalonia and the island of Mallorca, Spain).

## 2. Materials and Methods

### 2.1. Dogs and Sampling

The study was conducted in Camprodon (Catalonia, Spain) in July 2015 (n=14) and island of Mallorca (Balearic Islands, Spain) in February 2017 (n=10), both endemic areas of CanL. Twenty-four apparently clinically healthy outdoors dogs of both sexes (eleven females and thirteen males) were enrolled. Age ranged between 8 month and 9 years and the median was 3.5 years old. Veterinarians subjected all dogs to physical examination and found that they did not present any clinical signs and were apparently healthy with the exception of three dogs that presented only papular dermatitis due to* L. infantum*, a mild form of clinical leishmaniosis with commonly good prognosis in dogs [39].

Collection of blood samples for this study was performed in accordance with veterinary protocols under aseptic conditions. Approximately 7-10 mL of blood from jugular or cephalic venipuncture was collected and immediately transferred into sterile tubes divided into: 1 tube with sodium heparin (6 mL volume) for whole blood assay, 1 tube with ethylenediaminetetraacetic acid (EDTA) (2mL volume), and 1 tube for serum with clotting accelerator (2-4 mL volume) for* L. infantum *specific antibodies and hematology profile. Blood sampling was obtained as screening for* L. infantum *infection and for checking general health status.

The dog owners gave their consent for blood sampling. Detection of antibodies against* L. infantum* using an in-house quantitative enzyme linked immunosorbent assay (ELISA) was performed on sera of all dogs as previously described and blood* Leishmania* real-time polymerase chain reaction (RT-PCR) was performed in 21 out of 24 dogs as previously described [40].

### 2.2. Whole Blood Cytokine Release Assay

Heparinized whole blood cytokine release assay was performed as previously described [38]. Briefly, blood from Mallorca dogs (n=10) was stimulated as follows: (1) medium alone (*ɸ*), (2) medium with* L. infantum *soluble antigen (LSA) (provided by Dr. Cristina Riera,* Facultat de Farmacia*,* Universitat de Barcelona*) at a working concentration of 10 *μ*g/mL (*ɸ*+LSA), (3) medium with concanavalin A (ConA) a strong lymphocyte mitogen (Medicago® Uppsala, Sweden) at a working concentration of 10 *μ*g/mL (*ɸ*+ConA), (4) medium with TLR3a [Poly(I:C) Invivogen® San Diego, USA] at a working concentration of 10 *μ*g/mL (*ɸ*TLR3a), or TLR4a [Monophosphoryl lipid A (MPLA), Invivogen® San Diego, USA] at a working concentration of 1 *μ*g/mL (*ɸ*TLR4a), or TLR7a [Imiquimod-R837 (IMQ) Invivogen®, San Diego, USA] at a working concentration of 5 *μ*g/mL (*ɸ*TLR7a) and (5) medium with TLR3a, TLR4a or TLR7a at concentrations described above and LSA at a concentration described above (LSA+TLR3a or TLR4a or TLR7a). In the case of Catalonian dogs (n=14), blood was stimulated with all TLRa with the exception of TLR7a. All conditions were run in duplicate and incubated for 48 hours (h) and 5 days at 37°C in 5% CO_2_ enriched environment. After 48h and 5 days the samples were collected and centrifuged individually at 300 g for 10 minutes and the supernatants were collected and stored at -80°C until used. Cytokine concentrations were measured in supernatants collected at 48h for IL-6 and TNF-*α* and at 5 days for IFN-*γ*. Supernatants collected at 5 days for IFN-*γ* were only stimulated with medium, LSA and ConA.

### 2.3. Sandwich ELISA for Canine Cytokines

Cytokine analysis of IFN-*γ*, TNF-*α*, and IL-6 was performed according to manufacturer's instructions (DuoSet**®** ELISA by Development System R&D TM, Abingdon, UK) using 96-well plate flat bottom (Costar**®** Corning, New York, USA). Slight modifications were done for IFN-*γ* ELISA [24, 25]. Standard concentrations of IFN-*γ* started with 2000 pg/mL followed by twofold dilutions in reagent diluent (R&D systems, Minneapolis, USA) until 31.25 pg/mL was reached. Standard curve for TNF-*α* started with 1000 pg/mL and twofold dilutions were made until 15.6 pg/mL concentration. Standard curve for IL-6 started with 4000 pg/mL and twofold dilutions were made until 62.5 pg/mL concentration. All values under the last standard concentration were considered undetectable for each cytokine [24]. Duplicates of all supernatants studied were performed in all ELISAs. Optical density was measured with an ELISA reader (Anthos Reader 2020, Cambridge, UK) at wavelength of 450 nm. The standard curve for each cytokine was calculated using a computer generated four parameter logistic curve-fit with program myassays (http://www.myassays.com/). Plate was repeated when the R2-value of standard curve was below 0.98. Dogs were classified as IFN-*γ* producers when* L. infantum *specific IFN-*γ* concentration was detectable after subtracting medium alone. Dogs were classified as IFN-*γ* non-producers when* L. infantum *specific IFN-*γ* concentration subtracting medium alone was at undetectable levels [25].

### 2.4. Statistical Analysis

A nonparametric Wilcoxon signed rank test was used to compare among related several treatments. Differences were considered significant with a 5% significance level* (p<0.05).* The statistical analysis was performed using the SPSS 17.0 for Windows software (SPSS Inc., USA). Graph was performed using excel GraphPad Prism 7 (GraphPad Software, La Jolla, USA).

## 3. Results

### 3.1. Clinical Data

All 14 dogs from Catalonia were clinically healthy based on physical examination and hematological parameters. Furthermore, all dogs were seronegative and only four out of 14 dogs from Catalonia were low positive to RT-PCR (Table 1). There were nine males and five females with a median of age of 50.5 months and an age range from 12 months to 9 years. Four mixed Fox hounds, two Dachshunds, two Griffons nivernais, one mixed English pointer, two Arieges pointers, one Ibizan hound, one English setter, and one Bruno Jura hound were studied.

Seven out of 10 dogs from island of Mallorca were seronegative for* L. infantum* antibodies and three were low positive, two of them with papular dermatitis and one without any lesion. However, all dogs tested (n=7) resulted negative to blood RT-PCR. The remaining three dogs were not tested due to lack of sample. There were six females and four males. All ten dogs were Ibizan hounds. The median of age was 30 months with a range from 8 months to 6 years.

### 3.2. Specific* L. infantum* Antibody Levels, Blood RT-PCR, and IFN-*γ* Production

The results from specific* L. infantum* antibody levels, IFN-*γ* production, and blood RT-PCR from each individual dog tested (n=24) are summarized in Table 1.

Clinically healthy dogs (n=24) presented a mean ±standard deviation (mean ± SD): 16.6 ± 21.6 ELISA units (EU) of specific* L. infantum* antibody levels.

**Figure 1:**
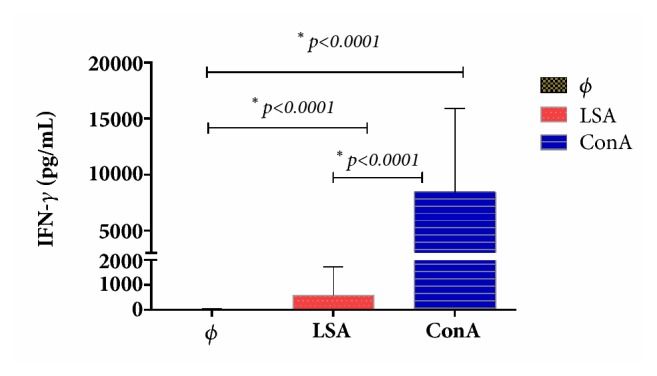
LSA specific IFN-*γ* production in blood* ex vivo *after LSA and ConA stimulation in all dogs studied. Φ: medium alone; LSA*: Leishmania* soluble antigen; ConA: concanavalin A; IFN-*γ*: Interferon-gamma.

Levels of IFN-*γ* of all animals under study (n=24) after LSA stimulation (mean ± SD of 584.5 ± 1131.4 EU) and ConA stimulation (mean ± SD of 8433.2 ± 7457.1 EU) were significantly higher than medium alone (mean ± SD of 12.0 ± 29.3 EU). The results from IFN-*γ* production at 5 days after stimulation with LSA and ConA from all dogs studied are shown in Figure 1. Additionally, fourteen (58.3%) out of twenty-four dogs were classified as parasite-specific IFN-*γ* producers (Table 1).

### 3.3. TNF-*α* Concentration

The results of TNF-*α* concentration for each treatment condition in all dogs studied (n=24) are summarized in Table 2. There was a significant increase of TNF-*α* production in samples supplemented with TLR4a* (p=0.0001)* and TLR7a* (p=0.005)* when compared with TLR3a in all dogs studied and also on TLR4a* (p=0.0001)* and TLR7a* (p=0.005)* in combination with LSA when compared with LSA+TLR3a. Concentration of TNF-*α* of all animals studied was significantly higher for all treatments [*ɸ*+LSA* (p*=*0.005)*,* ɸ*+ConA* (p*=*0.0001)*,* ɸ*+TLR3a* (p*=*0.044)*,* ɸ*+TLR4a* (p*=*0.0001)*,* ɸ*+TLR7a* (p=0.005),* LSA+TLR3a* (p*=*0.025),* LSA+TLR4a* (p*=*0.0001*), and LSA+TLR7a (*p=0.005*)] when compared to medium alone. Also,* ɸ*+TLR4a* (p=0.0001)* and* ɸ*+TLR7a* (p=0.013)* highly stimulated the production of TNF-*α* when compared to LSA alone. Moreover, higher production of TNF-*α* was observed after stimulation with LSA+TLR4a* (p*=*0.0001*) and LSA+TLR7a* (p*=*0.005*) compared to LSA alone. Also, after LSA+TLR3a stimulation levels of TNF-*α* were higher than* ɸ*+TLR3a stimulation* (p*=*0.052*).

### 3.4. IL-6 Concentration

The results of IL-6 concentration for each condition in all dogs studied (n=24) are summarized in Table 2. As observed with TNF-*α*, a significant increase of IL-6 production in TLR4a* (p=0.001)* and TLR7a* (p=0.005)* stimulated blood was found when compared with TLR3a in all dogs studied and also in TLR4a* (p=0.001)* and TLR7a* (p=0.005)* stimulated blood in combination with LSA compared with LSA+TLR3a. Concentration of IL-6 of all animals studied was significantly higher after* ɸ*+ConA* (p*=*0.019)*,* ɸ*+TLR4a* (p=0.0001)*,* ɸ*+TLR7a* (p=0.008*), LSA+TLR4a* (p*=*0.0001*), and LSA+TLR7a* (p=0.005)* treatments when compared to medium alone. Also,* ɸ*+TLR4a* (p=0.0001)* and* ɸ*+TLR7a* (p=0.007*) highly stimulated the production of IL-6 when compared to LSA alone. Moreover, higher production of IL-6 was observed after LSA+TLR4a* (p=0.0001*) and LSA+TLR7a* (p=0.005*) compared to LSA alone.

## 4. Discussion

The present results indicated for the first time a pronounced effect of TLR4a and TLR7a on cytokine production in blood from apparently healthy dogs living in endemic areas of CanL suggesting an active role of the innate and proinflammatory immune responses induced by these TLRa when compared with other TLRa or treatment conditions. In contrast, stimulation of TLR3a seems to have a less marked effect on cytokine production, supported by a statistically significant smaller production of proinflammatory cytokines when compared with TLR4a and TLR7a.

Improvement of vaccines and treatment strategies is imperative against leishmaniosis. In this context, new adjuvants are required to elicit an intense immune response needed for protection [41]. In particular, the vaccine adjuvant properties of imiquimod (TLR7a) and resiquimod (TLR7/8a) have been previously studied [42, 43]. Interestingly, very promising results were obtained from dogs stimulated blood with a synergic proinflammatory effect of TLR7a when combined with LSA, reflected in a higher production of TNF-*α*. A similar synergic effect of TLR2a in Ibizan hounds was previously observed but it was slightly less intense that the one observed here for TLR7a [38].

In agreement with the effect of TLR7a associated with LSA in this study, a previous* L. major *mouse model study described TLR7a and/or TLR7/8a as adjuvants [44]. It was found that* Leishmania* antigen alone was not protective to subsequent challenge unless administrated with TLR7/8a [44]. According to the synergic effect observed in the present study, Emami et al. detected an inferior Th1 response induced in Balb/c mice by TLR7a nanoliposomal immunization alone when compared to LSA plus TLR7a formulations, suggesting that TLR7a did not function well in the absence of antigen [45]. Contrarily, another study in a murine model showed that TLR7/8a seems to decrease/inhibit cytokine production induced by LSA [46]. Therefore, TLR7a appear to be good candidates for use as adjuvants for vaccines and immunotherapy in dogs. On the other hand, several studies performed in murine models and in humans showed promising results for the use of TLR7a as treatment in cutaneous [47] and visceral leishmaniasis [34]. Further studies are needed in order to characterize the therapeutic role of TLR7a in CanL.

Ligands of TLR4 have been broadly used in human vaccines as adjuvants and are the only TLRa approved due to their safety and the potent enhancement of the immune response [48]. In the present study, a marked cytokine production was observed after blood stimulation with a TLR4a alone and also when associated with LSA, supporting the role of TLR4 ligands as adjuvant. Recently, several studies have evaluated the use of TLR4a as adjuvant during* Leishmania* infection. Particularly, a vaccine antigen formulated with the TLR4a Glucopyranosyl lipid adjuvant in stable emulsion (GLA-SE) proved to induce antigen-specific responses that protected mice against* Leishmania donovani* infection and also demonstrated that prophylactic mouse immunization resulted in a marked reduction of the parasite spleen burden together with induction of memory CD4+ T lymphocytes enhancing IFN-*γ*, TNF-*α*, and IL-2 production [49]. Moreover, an innovative virus-like particles (VLP) vaccine loaded with sand fly saliva antigen plus* Leishmania *antigens and GLA-SE as adjuvant improved both cellular and humoral immune responses in a murine model and also in human peripheral blood mononuclear cells (PBMCs) [50].

In agreement with the results presented here, a previous study revealed a significant downregulation of several TLRs including TLR4 during disease progression in lymph nodes of experimentally infected dogs [51] suggesting a role of TLR4 in controlling infection and protect animals as shown in murine models of* L. major* infection [33, 52]. In contrast, so far, TLR4 transcription in unstimulated blood appears to be similar in healthy dogs and in dogs with different clinical stages of CanL at the time of diagnosis as well as during treatment follow-up [53, 54]. Further studies are warranted to elucidate the role of TLR4 in dogs in different clinical conditions and tissues.

The higher production of TNF-*α* and IL-6 after TLR4a blood stimulation when compared to untreated blood is in agreement with several studies carried out in murine and human leishmaniasis where TLR4 regulated the initial proinflammatory response [33, 55]. Additionally, and as observed by others [56], stimulation with LSA+TLR4a showed an augmentation in TNF-*α* production when compared with LSA stimulus alone. Particularly, a similar study to the one presented here revealed that activation of TLR4 by Lipopolysaccharide (LPS) augments TNF-*α* production by human PBMCs previously exposed to* L. braziliensis *amastigotes, suggesting a crucial role for TLR4 in TNF-*α* induction and subsequently in controlling leishmanial infection [56]. Similar results were observed in a murine model of* L. major* immunization where LSA ability to reduce parasite load was statistically significantly increased when associated with TLR4a [45, 46]. In view of the previous information together with the results presented here, TLR4 ligands should be also considerate in future studies as adjuvant for vaccines and immunotherapy in dogs.

In the present study, the effect of cytokine production of TLR3a was significantly less pronounced when compared with TLR4a and TLR7a. Interestingly, all dogs showed higher production of TNF-*α* after stimulation with TLR3a associated with LSA when compared with medium alone and with TLR3a. Recently, the essential role of TLR3a in inducing a Th1 response as adjuvant was demonstrated in a murine model of a* Leishmania amazonensis* vaccine based on total antigenic extract derived from promastigotes [57]. Therefore, TLR3a demonstrated its safety and ability to induce protection and its potential for being a promising candidate for* Leishmania *vaccines development. In contrast, other studies in which* Leishmania* RNA virus-1 (LRV1) was used to bind and stimulate endosomal TLR3 pathways in a murine model of* Leishmania guayanensis* infection confirmed that TLR3 is the dominant route of inflammatory pathology in this particular model [58, 59]. Further research conducted on* L. infantum* infection in dogs is necessary to elucidate the potential use of TLR3a as vaccine adjuvant or immunotherapy in sick dogs.

TNF-*α* is a powerful proinflammatory cytokine that promotes together with IFN-*γ* macrophage activation which is crucial for* Leishmania* parasite control both in dogs [60] and humans [18, 61]. However, little has been investigated on the function of TNF-*α* and IL-6 in dogs with clinical leishmaniosis. Moreover, less information is available related to dogs living in endemic areas of CanL and how these responses could affect the outcome of the disease in infected/exposed dogs. In the present study, a higher TNF-*α* response to LSA was observed when compared to medium alone in all dogs studied while this was not noted for IL-6, suggesting a more important role of TNF-*α* in inducing a proinflammatory response to* Leishmania *parasite. Accordingly, in previous studies, the use of TNF-*α* antagonists in subclinically infected people promoted the reactivation of infection by* Leishmania *spp. and the emergence of clinical signs of human leishmaniasis [62–64].

## 5. Conclusion

These findings indicate that TLR4a and TLR7a have a marked effect on cytokines production in* ex vivo *blood from apparently healthy dogs living in endemic areas of CanL. These data suggested an active role of TLRs on proinflammatory immune responses, which might be strongly involved in the process of disease resolution in dogs. In contrast, TLR3a seems to have a less pronounced effect on cytokine production in the present* ex vivo* model. Results presented here encourage further studies in order to evaluate the potential use of TLRa as vaccine adjuvant or as immunomodulatory therapy in* Leishmania* infection in dogs.

## Figures and Tables

**Table 1:** Clinical, serological, molecular, and parasite specific IFN-*γ* production from each individual dog tested (n=24).

**N**°	**SIGNALMENT**			**LSA IFN-** **γ** ** (pg/mL)** **(INTERPRETATION)**		**ELISA UNITS** **(INTERPRETATION)**		**RT-PCR** **(parasite/mL)** **(INTERPRETATION)**	
	**AGE (MONTHS)**	**BREED**	**SEX**						
1	96.0	FOX HOUND mixed	M	0.0	(NP)	5.2	(-)	0.5	(+)
2	24.0	FOX HOUND mixed	F	993.6	(P)	4.4	(-)	0.0	(-)
3	60.0	ARIEGE POINTER	F	10.7	(NP)	4.1	(-)	0.0	(-)
4	24.0	FOX HOUND mixed	M	0.0	(NP)	2.9	(-)	0.0	(-)
5	60.0	FOX HOUND mixed	M	41.5	(P)	4.2	(-)	0.0	(-)
6	72.0	GRIFFON NIVERNAIS	M	95.5	(P)	7.6	(-)	0.0	(-)
7	12.0	GRIFFON NIVERNAIS	M	314.2	(P)	7.9	(-)	0.5	(+)
8	48.0	ARIEGE POINTER	M	0.0	(NP)	6.1	(-)	0.2	(+)
9	72.0	IBIZAN HOUNDS	M	920.4	(P)	3.0	(-)	0.0	(-)
10	12.0	BRUNO DE JURA	F	0.0	(NP)	3.7	(-)	0.0	(-)
11	108.0	ENGLISH POINTER mixed	F	0.0	(NP)	10.1	(-)	0.0	(-)
12	48.0	DACHSHUNDS	M	10.0	(NP)	4.0	(-)	0.0	(-)
13	36.0	DACHSHUNDS	M	285.1	(P)	5.1	(-)	0.0	(-)
14	36.0	ENGLISH SETTER	F	142.9	(P)	4.9	(-)	0.7	(+)
15	48.0	IBIZAN HOUNDS	M	1635.0	(P)	81.4	(+)	0.0	(-)
16	36.0	IBIZAN HOUNDS	M	4663.0	(P)	34.5	(-)	0.0	(-)
17	24.0	IBIZAN HOUNDS	M	3196.0	(P)	16.6	(-)	0.0	(-)
18*∗*	8.0	IBIZAN HOUNDS	F	619.5	(P)	78.9	(+)	0.0	(-)
19*∗*	8.0	IBIZAN HOUNDS	F	14.8	(NP)	19.3	(-)	0.0	(-)
20*∗*	8.0	IBIZAN HOUNDS	F	0.0	(NP)	37.5	(+)	0.0	(-)
21	24.0	IBIZAN HOUNDS	F	0.0	(NP)	10.8	(-)	0.0	(-)
22	24.0	IBIZAN HOUNDS	F	119.7	(P)	21.3	(-)	N/D	
23	72.0	IBIZAN HOUNDS	F	574.5	(P)	10.6	(-)	N/D	
24	48.0	IBIZAN HOUNDS	M	165.9	(P)	16.8	(-)	N/D	

*∗*Dogs that presented stage I papular dermatitis.

LSA: *Leishmania* soluble antigen, IFN-*γ*: Interferon-gamma, pg: picograms, mL: milliliter, ELISA: enzyme linked immunosorbent assay, RT-PCR: real time polymerase chain reaction, M: male, F: female, NP: non-IFN-*γ* producer, P: IFN-*γ* producer, (-): negative, (+): positive, and N/D: no data.

**Table 2:** Results of TNF-*α* and IL-6 concentrations after stimulation at each condition in all dogs studied (n=24).

**Cytokines (pg/mL)**	**Treatment conditions (mean ± SD)** **∗**								
	Φ	ɸ**+LSA**	ɸ**+ConA**	ɸ**+TLR3a**	ɸ**+TLR4a**	ɸ**+TLR7a**	**LSA+TLR3a**	**LSA+TLR4a**	**LSA+TLR7a**
**TNF-** **α**	9.6±17.1	27.1±32.7	107.7±107.7	17.2±28.0	116.3±10.9	92.0±56.1	18.8±17.2	116.1±73.8	157.1±78.1
**IL-6**	15.1±19.5	12.6±19.0	24.3±22.7	17.0±26.8	76.7±39.9	55.1±32.2	16.9±20.4	80.6±39.3	75.4±39.7

*∗*Mean ±standard deviation (mean ± SD), medium alone (Φ); *Leishmania* soluble antigen (LSA); Concanavalin A (ConA); TLR3 [Poly(I:C)] agonist (Φ+TLR3a) and TLR3a and LSA (LSA+ TLR3a); TLR4 (MPLA) agonist (Φ+TLR4a) and TLR4a and LSA (LSA+ TLR4a); TLR7 (IMQ) agonist (Φ+TLR7a) and TLR7a and LSA (LSA+ TLR7a)

## Data Availability

The data that support the findings of this study are available from the corresponding author, Laia Solano Gallego (LSG) upon reasonable request.

## Abreviations

CanL: Canine leishmaniosis
CO_2_: Carbon dioxide
CD4: Cluster of cuadruple differentiation
CMI: Cell-mediated immune
ConA: Concavalin A
DC: Dentritic cells
EDTA: Ethylenediaminetetraacetic acid
ELISA: Enzyme-linked immunosorbent assay
GLA-SE: Glucopyranosyl lipid adjuvant in stable emulsion
IFN-*γ*: Interferon gamma
IL-12: Interleukin 12
IL-6: Interleukin 6
IMQ: Imiquimod-R837
iNos: Nitric oxide synthase
LPG: Lipophosphoglycan
LPS: Lipopolysaccharide
LRV1: * Leishmania* RNA virus-1
LSA: * Leishmania* soluble antigen
mL: Milliliters
MPLA: Monophosphoryl lipid A
NF-*κ*B: Nuclear factor kappa-light-chain-enhancer of activated B cells
NK: Natural killers
nm: Nanometers
NO: Nitric oxide
PBMCs: Peripheral blood mononuclear cells
PAMPs: Pathogen-associated molecular patterns
PRRs: Patter recognition receptors
ROS: Reactive oxygen species
SD: Standard deviation
Th1: T helper 1
Th2: T helper 2
TLRs: Toll-like receptors
TLR3: Toll-like receptors 3
TLR4: Toll-like receptors 4
TLR4: Toll-like receptors 7
TLRa: Toll-like receptor agonists
TNF-*α*: Tumor necrosis factor alpha
VL: Visceral leishmaniosis
VLP: Virus-like particles
Φ: Medium alone.
